# Using non-contrast-enhanced magnetic resonance venography for the evaluation of May-Thurner syndrome in patients with renal insufficiency: A case report

**DOI:** 10.1097/MD.0000000000018427

**Published:** 2019-12-27

**Authors:** Yin-Chen Hsu, Yao-Kuang Huang, Li-Sheng Hsu, Pang-Yen Chen, Chien-Wei Chen

**Affiliations:** a Department of Diagnostic Radiology; b Chang Gung University College of Medicine, Taoyuan; c Division of Thoracic and Cardiovascular Surgery, Wound Center and Plastic Surgery, Chang Gung Memorial Hospital Chiayi Branch, Chiayi; d Department of Biomedical Engineering, National Cheng Kung University, Tainan; e Department of Emergency Medicine, Mackay Memorial Hospital, Taipei; f Institute of Environmental and Occupational Health Sciences, National Yang-Ming University; g Institute of Medicine, Chung Shan Medical University, Taichung, Taiwan.

**Keywords:** 3D-TSE, chronic renal insufficiency, contrast-induced nephropathy, May-Thurner syndrome, non-contrast-enhanced MRV

## Abstract

**Rationale:**

Contrast-enhanced computed tomographic venography (CTV) or magnetic resonance venography (MRV) are usually used to detect May-Thurner syndrome (MTS). However, both are associated with contrast-induced nephrotoxicity. For patients who cannot receive contrast media, non-contrast-enhanced MRV using three-dimensional (3D) turbo spin-echo (TSE) is considered an alternative. We report a case of MTS to describe its clinical utility and advantages.

**Patient concerns:**

A 49-year-old male experienced isolated left leg swelling and pain for half a month. He had a history of chronic renal insufficiency that made contrast-enhanced imaging studies inadequate.

**Diagnoses:**

A lower extremity venous Duplex scan showed a thrombus extending from the left distal femoral vein to the popliteal vein with valvular reflux, consistent with infrainguinal deep vein thrombosis (DVT). The suprainguinal DVT was evaluated by non-contrast-enhanced MRV. The results showed sandwich external compression of the left common iliac vein between the right common iliac artery and lumbar vertebrae, consistent with DVT of the left common iliac vein caused by MTS.

**Interventions:**

The patient received angioplasty with the implantation of a balloon-expandable stent over the left common iliac vein.

**Outcomes:**

Excellent recanalization of the left iliac vein was noted postoperatively.

**Lessons:**

In the evaluation of suprainguinal venous lesions, non-contrast-enhanced MRV presents the venous structure alone at high resolution without the accompanying arterial structure, which makes it an excellent diagnostic imaging tool for MTS. These findings indicate that non-contrast-enhanced MRV could be useful for detecting systemic venous pathologies in patients with renal insufficiency.

## 1 Introduction

May-Thurner syndrome (MTS) is defined as an anatomic variant of right common iliac artery compression of the left common iliac vein with subsequent partial or complete impedance of the iliac vein outflow.^[1]^ When determination of the thrombus extent is necessary, contrast-enhanced computed tomographic venography (CTV), or magnetic resonance venography (MRV) may be used. Despite the advantages of each diagnostic tool, patient safety and the availability of these tools are significant considerations.[^2^,^3^] For patients suspected of MTS who cannot receive contrast media, non-contrast-enhanced MRV using three-dimensional (3D) turbo spin-echo (TSE) is considered an alternative modality.

We report a case of MTS with a comorbidity of chronic renal insufficiency and describe the clinical utility and advantages of non-contrast-enhanced MRV. To the best of our knowledge, the application of this modality for diagnosing MTS has not been reported previously.

## 2 Case presentation

A 49-year-old male presented with isolated left leg swelling and pain. The patient was concerned because the symptoms had progressed over the course of 2 weeks. He reported no recent trauma, prolonged immobilization, or bed rest, and had not experienced any weakness, paresthesia, pallor, or coldness associated with the left leg. He had a history of chronic renal insufficiency, recurrent deep vein thrombosis (DVT), and pulmonary embolism. On physical examination, left lower extremity examinations revealed severe swelling with +2 pitting edema extending from the thigh to the ankle and moderate tenderness of the left leg. The right lower extremity was normal on examination. Blood tests revealed normal hemogram with a creatinine level of 3.85 mg/dl.

**Figure 1:**
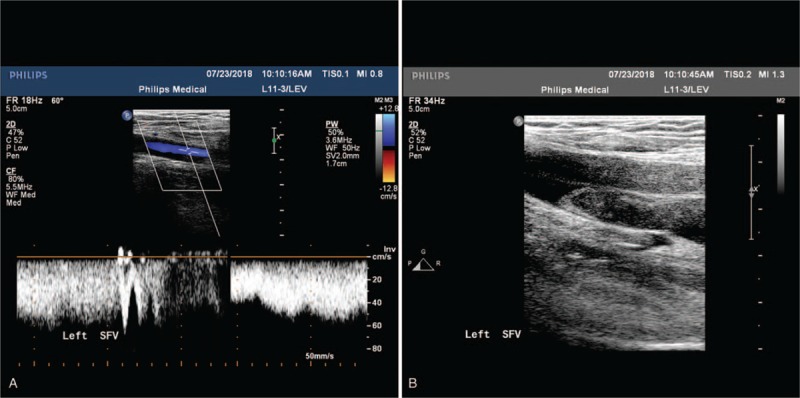
Infrainguinal venous Duplex ultrasonography findings. (A) Valvular reflux in the left superficial femoral vein (SFV). (B) Venous thrombus in the left SFV.

**Figure 2:**
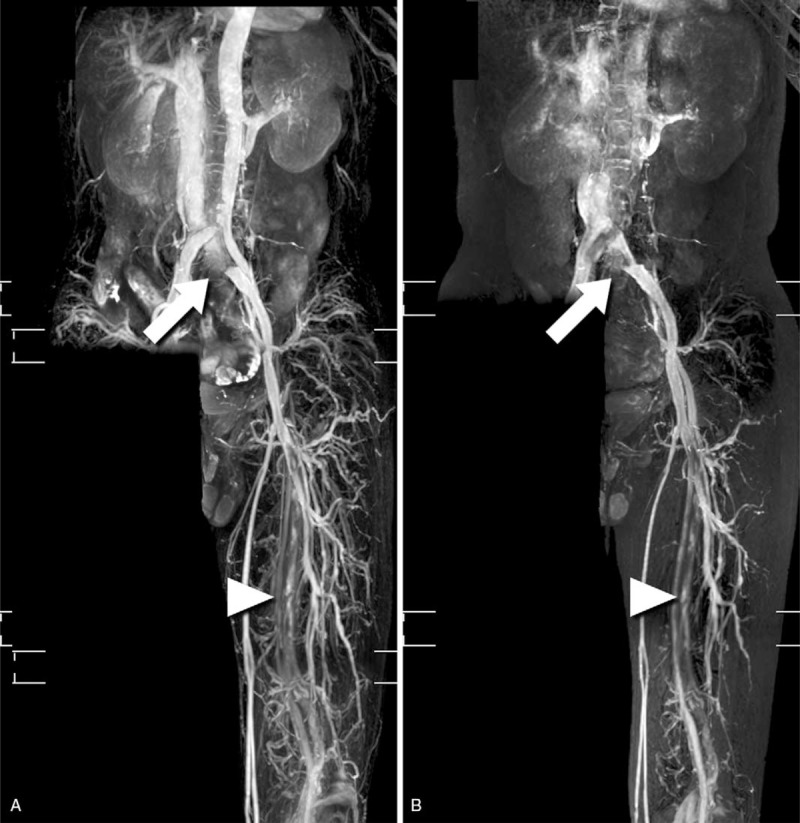
Non-contrast-enhanced magnetic resonance imaging (MRI) using three-dimensional (3D) turbo spin-echo (TSE) with cardiac triggering. (A) For imaging arteries and veins, a 3D-TSE protocol with short tau inversion recovery (STIR) is scanned with diastolic triggering. (B) To obtain veins alone, a 3D-TSE protocol with STIR is scanned with systolic triggering. Sandwich external compression of the common iliac vein (CIV) between the right common iliac artery and lumbar vertebrae (arrow). Flow signal loss of left SFV (arrowhead), indicated infrainguinal deep venous thrombosis.

**Figure 3:**
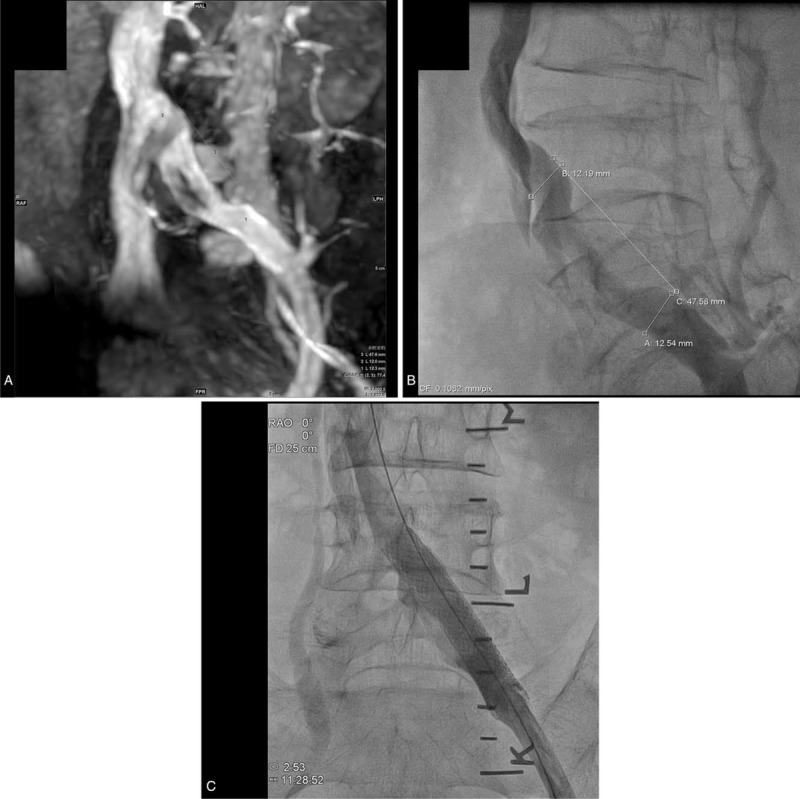
Angioplasty using balloon-expandable stent. (A) Non-contrast-enhanced MRV showed segmental narrowing measuring 12.0 × 47.6 mm. (B) Preoperative venography showed segmental narrowing measuring 12.2 × 47.6 mm, close to the MRV measurement. (C) The balloon-assisted stent (14 × 60 mm) deployment was then performed to accurately cover the segmental narrowing. Postoperative venography showed patency of the left CIV.

A lower extremity venous Duplex scan showed a thrombus extending from the left distal femoral vein to the popliteal vein with valvular reflux, consistent with infrainguinal DVT (Fig. 1). Considering the risk of renal toxicity, the suprainguinal DVT was evaluated by non-contrast-enhanced MRV. The results showed sandwich external compression of the left common iliac vein between the right common iliac artery and lumbar vertebrae, consistent with DVT of the left common iliac vein caused by MTS (Fig. 2). Venography with injection via the left common femoral vein showed a left common iliac vein obstruction with external compression (Fig. 3A-B). Therefore, angioplasty with the implantation of a balloon-expandable stent over the left common iliac vein was performed. Excellent recanalization of the left iliac vein was noted postoperatively with venography (Fig. 3C).

The Institutional Review Board (IRB) of Chang Gung Memorial Hospital approved this study (IRB number: 201900448B0). Written informed consent for publication from study participant was obtained for the study.

## 3 Discussion

MTS is an anatomically variable condition of extrinsic venous compression of the vertebral bodies by the arterial structure.^[4]^ Although the syndrome is rare it may cause subsequent extensive DVT of the ipsilateral extremity, resulting in severe disability and post-thrombotic syndrome. The actual incidence of MTS is unknown and is likely to be underestimated.^[5]^ Any patient with left iliac vein thrombosis should be investigated for suspected MTS and work-up studies should be performed if indicated.^[6]^

A diagnosis of MTS can be made with a combination of the clinical presentation and imaging studies. Venous Duplex ultrasonography for the identification of iliofemoral thrombus is generally considered as the first step in the diagnosis of MTS. It allows evaluation of the functional and dynamic status of the involved vein; however, the limited visualization of the iliac veins in certain patients may reduce its reliability.[^7^,^8^]

Cross-sectional imaging modalities are usually used for further evaluation of the iliac venous pathology. Ideally, both CTV and MRV with a contrast medium can clearly identify the stenotic lesions and specific causes of extrinsic venous compression that contribute to the symptoms.^[9]^ However, the major challenge faced by both imaging modalities is the difficulty in estimating the proper acquisition time for optimal contrast opacification of the target vein.^[10]^ This is a universal technical issue for contrast-enhanced vascular imaging modalities, and it becomes trickier in the evaluation of the iliac venous system. There are intrinsic patient factors (e.g., cardiac output) and an extrinsic compression mechanism that can alter the contrast transit time, to various degrees.[^10^,^11^,^12^] The net result is an inadequate acquisition time, which limits the image resolution and makes detection of subtle lesions difficult. For those patients with a risk of contrast-induced nephrotoxicity, it is not considered worth the trade-off to receive contrast-enhanced CTV or MRV, to exclude obstructive iliac vein lesions.

Utilizing 3D-TSE techniques with the short tau inversion recovery protocol, non-contrast-enhanced MRV has the ability to suppress static background tissue and eliminate arterial signals. This sequence facilitates an angiographic image, which presents only the high-resolution venous structure without contamination by the accompanying artery. It provides an excellent signal-to-noise ratio and produces vivid blood images, which make it an excellent diagnostic imaging tool for obstructive iliac venous pathology. More importantly, this modality is particularly suitable for patients who are vulnerable to contrast agents. This study was conducted with a clinical case presentation. When generalizing the situations and findings, further large-scale studies are required to explore the validity and reliability of non-contrast-enhanced MRV.

In conclusion, non-contrast-enhanced MRV can present isolated venous structures with high-resolution due to its excellent background suppression ability. This modality provides robust power in vascular imaging that is simultaneously free of radiation and contrast agent toxicity. Therefore, non-contrast-enhanced MRV can be a useful tool in the diagnosis of venous diseases such as MTS in patients with renal insufficiency.

## Author contributions

**Conceptualization:** Yin-Chen Hsu, Chien-Wei Chen.

**Data curation:** Yin-Chen Hsu, Chien-Wei Chen.

**Writing – original draft:** Yin-Chen Hsu.

**Writing – review & editing:** Yao-Kuang Huang, Li-Sheng Hsu, Pang-Yen Chen, Chien-Wei Chen.

Yin-Chen Hsu orcid: 0000-0003-4483-486X.
